# Genome-Wide Dissection of the TCP Gene Family in Peach and Expression Analysis Under Drought Stress

**DOI:** 10.3390/ijms27156860

**Published:** 2026-07-30

**Authors:** Yanfu Jing, Yang Yu, Zimin Xiao, Yaoguang Xu, Yu Tian, Yanyan Su, Hua Xie

**Affiliations:** 1 Institute of Forestry and Pomology, Beijing Academy of Agriculture and Forestry Sciences, Beijing 100093, China; jingyanfu92@163.com (Y.J.); yuyangff@hotmail.com (Y.Y.); xiaozm13651457048@163.com (Z.X.); b040381@126.com (Y.X.); tianyu@baafs.net.cn (Y.T.); suyanyan5566@163.com (Y.S.); 2 Beijing Engineering Research Center for Deciduous Fruit Trees, Beijing 100093, China; 3 Key Laboratory of Biology and Genetic Improvement of Horticultural Crops (North China), Ministry of Agriculture and Rural Affairs, Beijing 100093, China

**Keywords:** peach, TCP gene family, expression profiling, drought stress, subcellular localization

## Abstract

The TCP (*Teosinte branched* 1/*Cycloidea/Proliferating*) gene family comprises plant-specific transcription factors essential for regulating growth, development, and environmental adaptation. Utilizing the high-quality ‘Rui Youpan 1’ (‘RYP1’) reference genome, we conducted a comprehensive genome-wide identification and characterization of the TCP family in peach (*Prunus persica*). We identified 20 *PpTCP* genes and systematically evaluated their physicochemical properties, chromosomal distribution, phylogeny, gene architecture, and promoter *cis*-regulatory elements. Notably, the enrichment of abscisic acid-responsive elements (ABREs) in their promoters suggests a significant role in abiotic stress signaling. Integrated RNA-seq and RT-qPCR analyses identified six putative candidates (*PpTCP3*, *5*, *6*, *11*, *14*, and *15*) with pronounced differential expression under drought conditions. Subcellular localization confirmed that all tested PpTCPs function within the nucleus. Moreover, the results of STRING-based computer simulations predicting protein–protein interactions indicate that PpTCP3 and PpTCP5 interact with key hormone pathways and stress-related transcription factors (TFs), including auxin signaling and strigolactone signaling.

## 1. Introduction

Peach (*Prunus persica* (L.) Batsch) is a globally vital temperate fruit crop, with the 2022 global production exceeding 25 million tons and a market valuation surpassing $22.5 billion USD in 2022 according to data from the Food and Agriculture Organization of the United Nations. Originating in China over two million years ago, the species possesses a rich evolutionary history and diverse germplasm [1]. Recent breakthroughs in high-throughput sequencing have yielded several high-quality genome assemblies [2,3,4,5], establishing peach as a premier model for Rosaceae functional genomics. While fruit sweetness, governed primarily by sucrose, which constitutes approximately 75% of soluble sugars in peach, is a defining agronomic trait [6], it remains highly sensitive to environmental pressures. It is important to note that external drought conditions may have a detrimental effect on the sugar content of fruits, thereby impacting their quality. This has been observed in peach, smooth-pit peach, and pomegranates [7,8,9,10]. Consequently, we hypothesise that the analysis of the mechanisms underlying peach drought tolerance is imperative for maintaining stable yields and enhancing fruit quality.

The TCP gene family, named after its founding members *Teosinte branched 1*, *Cycloidea*, and *Proliferating cell nuclear antigen factor* [11,12,13], is classified into two major groups based on the conserved TCP domain: Class I (PCF or TCP-P) [14,15] and class II (TCP-C) [16]. These transcription factors serve as developmental switches [17,18,19,20] and stress integrators [21], including drought [22], salt stress [23], and low-temperature [24]. In *Arabidopsis*, TCP14 and TCP15 modulate axillary branching [25] and pollen wall integrity [26], while the TCP8/15/21/22/23 cluster regulates cell proliferation via *KNOX1* suppression [27]. Similarly, TCP16 is indispensable for early pollen development [28], a role mirrored by *GhCYC2* in gerbera [29] and *HaCYC2c* in sunflower [30]. In chrysanthemum (*Chrysanthemum morifolium*), the photoperiod-dependent flowering in chrysanthemum is modulated by a novel regulatory module in which *CmTCP7* functions as a floral repressor, and its upstream transcriptional regulator *CmARF3* promotes flowering by directly repressing *CmTCP7* expression [31]. In *Physalis*, the heterometric expression of an LBD gene via LBD-TCP assembly serves as a regulatory mechanism that governs the size of floral organs and the weight of fruits [32]. Beyond development, TCPs facilitate physiological adaptations. For instance, AtTCP3 modulates auxin biosynthesis by repressing *CYP83B1/SUR2* [33]. In rice, OsTCP21 acts as a negative regulator of cold signaling [34], whereas OsPCF2 enhances salinity and drought tolerance by directly activating the *OsNHX1* promoter [35].

The expansion of genomic data has allowed for the systematic identification of TCP families in various taxa, including *Cenchrus* [36], *Elymus sibiricus* [37], oat [38], *Chrysanthemum indicum* [39], *Betula platyphylla* [40], *Brassica napus L.* [41], *Vigna radiata* [42], *Pogostemon cablin* [5], and *Brassica juncea* [43]. While previous studies in peach have primarily examined TCP expression during fruit ripening [44], their involvement in the drought response remains poorly understood. In this study, we leveraged the ‘RYP1’ genome to re-evaluate the peach TCP family, providing a detailed analysis of their structural characteristics and transcriptional responses to drought. Our findings offer new insights into the regulatory networks governing drought resilience in peach and identify promising genetic targets for crop improvement.

## 2. Results

### 2.1. Identification and Chromosomal Distribution of Peach TCP Genes

**Figure 1:**
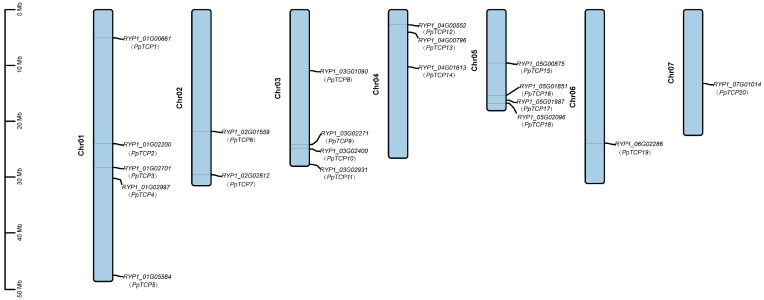
Chromosomal distribution of *PpTCP* genes in peach. Gene names on the right of each chromosome indicate their relative positions. The scale bar on the left denotes linkage group length in Megabases (Mb).

A genome-wide screening of the ‘RYP1’ peach genome identified 20 *TCP* genes, designated *PpTCP1* through *PpTCP20* based on their chromosomal positions (Table 1; Figure 1). Corresponding gene IDs in the LOVE II genome are provided in Table S1. Physicochemical analysis of the predicted proteins revealed amino acid lengths ranging from 192 (*PpTCP8*) to 603 residues (*PpTCP5*), with molecular weights spanning 24.77 kDa to 63.10 kDa (Table 1). Instability index values ranged from 43.90 (*PpTCP8*) to 74.06 (*PpTCP12*) and 14 of the 20 members exceeded a threshold of 50.00. While aliphatic indices were generally high, ranging from 51.44 (*PpTCP5*) to 86.16 (*PpTCP20*), all members exhibited a negative grand average of hydropathy (GRAVY) scores. This indicates a hydrophilic nature, consistent with their functional roles in aqueous environments such as the nucleoplasm [45,46,47].

Given that TCP transcription factors are typically nuclear-localized, we hypothesize that the *PpTCP* family operates within the nucleus [48,49]. These 20 genes are unevenly distributed across seven chromosomes (Figure 1). Chromosome 01 (Chr01) contains the highest density with five members (*PpTCP1–5*), followed by Chr03 and Chr05 with four genes each. Chr04 harbors three genes, Chr02 contains two, and Chr06 and Chr07 each possess a single member. No *TCP* genes were detected on Chr08, and notably, no tandem duplication events were observed. The diverse structural profile and asymmetrical distribution suggest that the *PpTCP* family has undergone significant evolutionary specialization or functional diversification within the peach genome.

### 2.2. Phylogenetic Classification of PpTCP Genes

**Figure 2:**
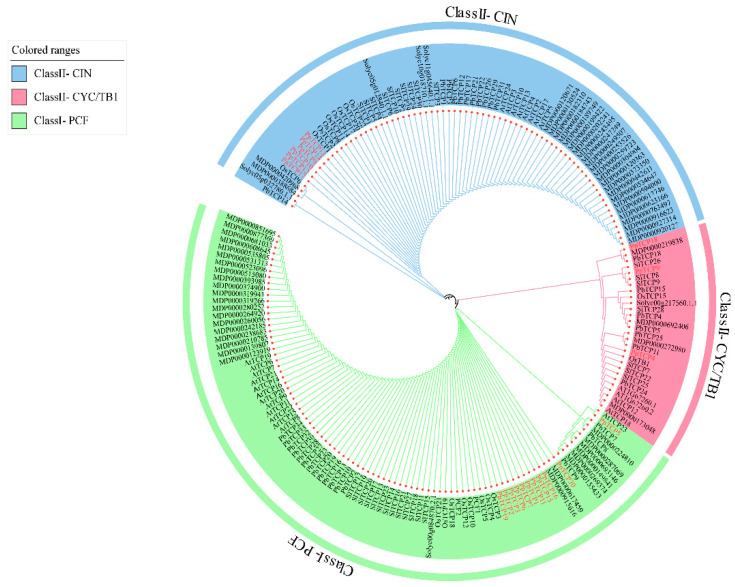
Phylogenetic analysis of TCP proteins across six plant species. Clades are color-coded: Class I (PCF), Class II-CIN, and Class II-CYC/TB1. The 20 peach proteins are highlighted in red. A Maximum Likelihood (ML) phylogenetic tree [50] was constructed using MEGA 7.0 [56] with 1000 bootstrap replicates.

To elucidate evolutionary relationships, a Maximum Likelihood phylogenetic tree was constructed using TCP protein sequences from *Arabidopsis thaliana*, *Oryza sativa*, *Solanum lycopersicum*, *Malus domestic*, *Pyrus bretschneideri* and *P. persica* (Figure 2) [50]. The 20 peach TCPs were categorized into three distinct clades: Class I-PCF (11 genes), Class II-CIN (6 genes), and Class II-CYC/TB1 (3 genes). The PCF clade is the largest, representing 55% of the family, which likely reflects its broad involvement in growth, development, and stress adaptation [11,51].

Evolutionary clustering revealed that *P. persica* is most closely related to *P. bretschneideri* (pear) and *M. domestica* (apple). For instance, PpTCP18 clustered tightly with apple MDP0000219838 and pear PbTCP18 within the CYC/TB1 clade [52]. The apple ortholog (annotated as *MD06G1211100* in the GDDH13 assembly) is a known homolog of *BRANCHED1* (*BRC1*), a key regulator that inhibits shoot branching [53]. Similarly, PpTCP9 showed high homology with tomato SlTCP8 and SlTCP9, both involved in lateral shoot regulation [54]. In the PCF clade, PpTCP5 clustered with AtTCP23, which is known to modulate flowering time and vegetative development [55].

### 2.3. Gene Architecture and Conserved Domains

**Figure 3:**
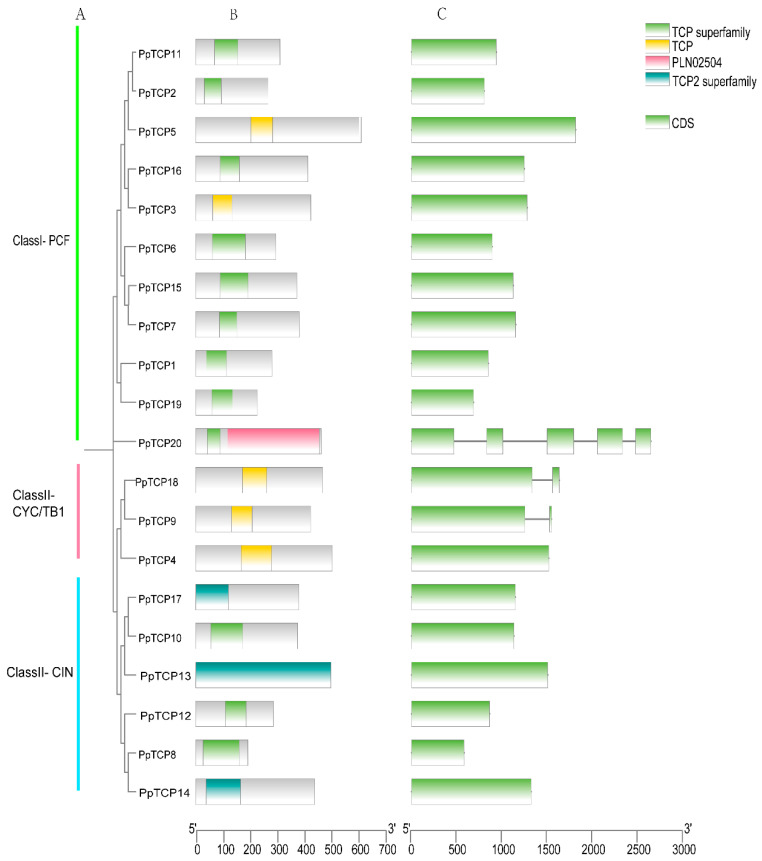
Structural and domain analysis of peach PpTCP proteins. (**A**) ML phylogenetic tree of PpTCPs. (**B**) Conserved domain architecture: TCP superfamily (green), core TCP domain (yellow), PLN02504 (pink), and TCP2 superfamily (blue). (**C**) Exon–intron organization; green boxes represent CDS regions.

The structural organization of the *PpTCP* family is illustrated in Figure 3. Phylogenetic grouping (Figure 3A) aligns with the multi-species analysis, confirming the division into PCF, CYC/TB1, and CIN clades. All identified members harbor the signature TCP or TCP2 superfamily domain, confirming their evolutionary conservation (Figure 3B).

Gene structure analysis (Figure 3C) showed that members within the same subfamily share highly similar exon–intron patterns. In total, 17 of the 20 *PpTCP* genes are intronless, consisting of a single coding sequence (CDS), a dominant trait in this family. This single-CDS architecture is strictly maintained in 10 members of the PCF clade and all 6 members of the CIN clade. The only exception in the PCF clade is *PpTCP20*, which contains five exons and four introns. Within the CYC/TB1 clade, *PpTCP4* follows the single-CDS model, while *PpTCP18* and *PpTCP19* each possess two CDSs, one of which appears truncated.

### 2.4. Sequence Alignment and Motif Composition of PpTCP Genes

**Figure 4:**
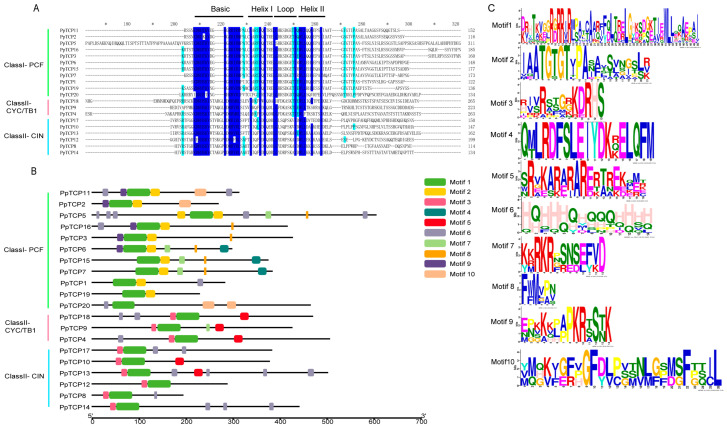
Structural and motif analysis of the *PpTCP* family. (**A**) Multiple sequence alignment highlighting the conserved bHLH-type TCP domains. The basic helix–loop–helix structure is indicated above the sequences. The asterisk (*) indicates every tenth amino acid position in the alignment. (**B**) Distribution of 10 conserved motifs within PpTCP proteins, with unique color-coded boxes representing each motif. (**C**) Logo analysis of identified motifs generated via MEME. Bit height represents the conservation level of each amino acid residue at specific positions. Protein sequence lengths are indicated by the scale bar. Comprehensive MEME parameters are detailed in the Section 4.

To define the structural characteristics of PpTCPs, we performed multiple sequence alignments of their deduced amino acids (Figure 4A). All members harbor a centrally located, putative TCP domain characterized by a basic helix–loop–helix (bHLH) architecture. Sequence conservation was notably higher within the PCF clade compared to the other two subgroups; specifically, PCF members exhibit a distinct four-amino acid deletion within the basic region (Figure 4A). The Helix I domain is enriched with conserved glycine (G) and phenylalanine (F) residues, while Helix II is defined by a high frequency of tryptophan (W) and leucine (L), the latter forming a characteristic LXXLL motif (Figure 4A).

Using the MEME suite, we identified 10 conserved motifs across the family (Figure 4B,C). Motif distribution patterns were largely clade-specific, reflecting evolutionary divergence. Motif 1, corresponding to the essential bHLH domain, is universally conserved across all 20 PpTCPs, underscoring its functional indispensability (Figure 4B,C). Conversely, motifs 2, 8, and 10 are unique to the PCF clade, though motif 2 was notably absent in PpTCP20. Within Class II, motifs 3 and 5 are restricted to the CYC/TB1 and CIN clades; while motif 3 is a shared hallmark of both, motif 5 is exclusive to the CYC/TB1 clade, appearing only sporadically in CIN members *PpTCP10* and *PpTCP13* (Figure 4B). Such variations in motif architecture suggest that these clades have likely diverged to fulfill specialized regulatory roles.

### 2.5. Promoter Cis-Element and Collinearity Analysis

**Figure 5:**
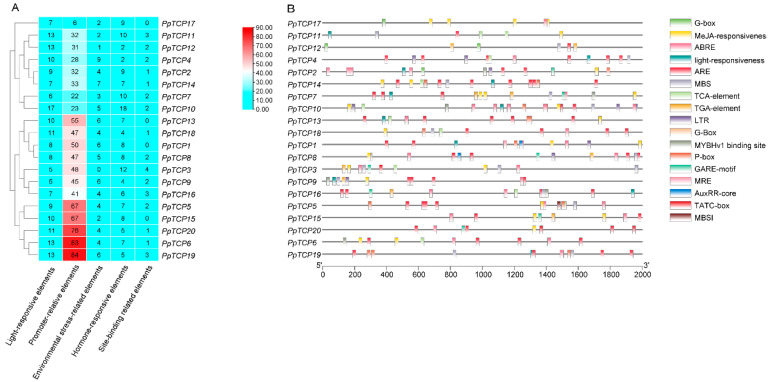
Analysis of *cis*-regulatory elements in *PpTCP* promoters. (**A**) Quantitative distribution of five distinct classes of *cis*-elements identified within each *PpTCP* promoter. (**B**) Map of non-light-responsive regulatory elements generated using TBtools v2.486 [59], illustrating the precise location, category, and abundance of motifs within the promoter regions.

To investigate the transcriptional regulation of *PpTCP* genes, we analyzed the 2000 bp promoter regions upstream of their start codons. The *PpTCP* promoters are enriched with *cis*-acting elements associated with light signaling, hormone sensitivity, and environmental stress (Figure 5A). Core transcriptional machinery, such as TATA and CAAT boxes, was present in all members, alongside diverse light-responsive motifs including Sp1, Gap-box, ACE, and GA-motifs (Figure 5A,B; Table S2). Among hormone-related elements, methyl jasmonate (MeJA)-responsive motifs (CGTCA and TGACG) were the most frequent, followed by abscisic acid (ABA)-responsive elements (ABREs) [57,58]. Although ABREs were not the most numerous, their broad distribution is significant, occurring in the promoters of 18 members (excluding *PpTCP12* and *PpTCP14*) (Figure 5A,B; Table S2). While the presence of these elements may suggest a potential involvement of the PpTCP family in hormone-mediated signaling, it is important to note that these are computational predictions that require experimental validation.

**Figure 6:**
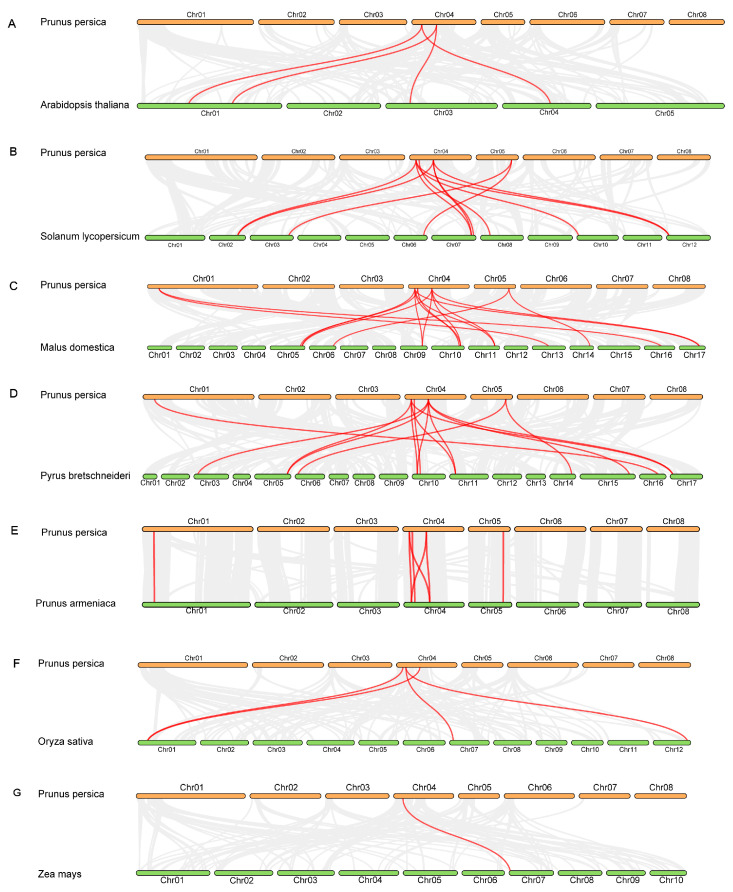
Synteny analysis of *TCP* genes between peach and seven representative species. Comparative genomics between *P. persica* and *A. thaliana* (**A**), *S. lycopersicum* (**B**), *M. domestica* (**C**), *P. bretschneideri* (**D**), *P. armeniaca* (**E**), *O. sativa* (**F**), and *Z. mays* (**G**). Chromosomes of *P. persica* are shown in orange, and chromosomes of other species are displayed in green. Gray lines indicate general syntenic blocks, while red lines highlight specific orthologous *TCP* gene pairs between peach and the reference genomes.

We further conducted a genome-wide collinearity analysis to trace the evolutionary history of *PpTCPs* relative to seven other species (*A. thaliana*, *S. lycopersicum*, *M. domestica*, *P. bretschneideri*, *Prunus armeniaca*, *O. sativa*, and *Z. mays*). Peach *TCPs* exhibited the highest synteny with *M. domestica* (17 orthologous pairs), followed by *P. bretschneideri* (14 pairs), *S. lycopersicum* (11 pairs) and *P. armeniaca* (7 pairs) (Figure 6), reinforcing the phylogenetic trends observed earlier. In contrast, only four orthologous pairs were identified with *A. thaliana* and *O. sativa*, and a single pair with *Z. mays*. This high degree of collinearity among *Rosaceae* species may be indicative of genomic positional conservation and evolutionary conservation among *Rosaceae* species. Notably, dicotyledonous plants showed substantially closer genomic affinity to peach than monocots. Interestingly, no collinear TCP pairs were identified on peach chromosomes 2, 3, 6, and 7 across the seven-species comparison.

### 2.6. Expression Profiling of PpTCP Genes Under Drought Stress

**Figure 7:**
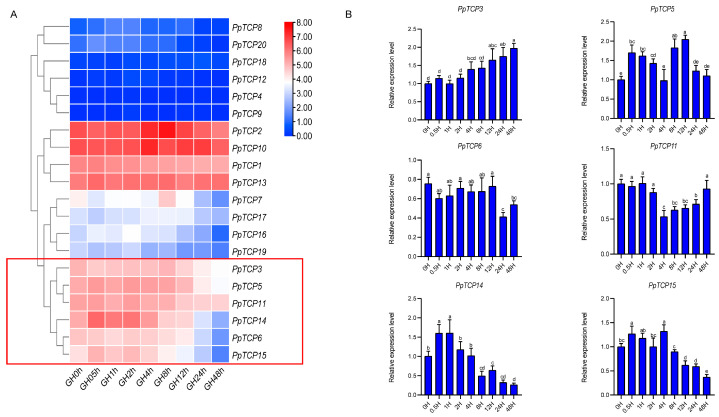
Transcriptional dynamics of *PpTCP* genes under drought stress. (**A**) Heatmap illustrating expression profiles of 20 *PpTCP* genes over a 48-h drought time-course (0, 0.5, 1, 2, 4, 8, 12, 24, and 48 h). The red box highlights six putative candidates (*PpTCP3*, *5*, *6*, *11*, *14*, and *15*) with significant expression shifts. (**B**) RT-qPCR validation of the six putative candidate genes. Relative expression was calculated using the 2^−△△Ct^ method, confirming the reliability of the transcriptomic data. Error bar represented the SD (n = 3). Different letters (a–e) indicate significant differences between means as determined by ANOVA followed by Duncan’s multiple range test (*p* < 0.05).

To determine the transcriptional dynamics of *PpTCPs* during water deficit, we integrated RNA-seq profiling with RT-qPCR validation. Transcriptomic analysis of peach fruits subjected to drought over a 48-h period revealed that, while the majority of the 20 *PpTCP* genes remained stable, a specific subset showed acute sensitivity [60]. *PpTCP3*, *5*, and *11* were rapidly induced during the early stages of stress before gradually declining, whereas *PpTCP6*, *14*, and *15* were progressively downregulated as stress prolonged (Figure 7A).

RT-qPCR validation of these six differentially expressed genes (DEGs) confirmed that their expression trends align closely with the RNA-seq data (Figure 7B). With the exception of *PpTCP3* and *5*, most tested genes showed significant downregulation by the 24-h mark, a trend most evident in *PpTCP14* and *15*. Notably, *PpTCP14* and *15* exhibited a sharp, transient peak just 0.5 h after the onset of stress. The robust and expeditious responses manifested by *PpTCP3*\*5*\*14*\*15* imply that these four genes may contribute to the response to external drought stress signals in peach.

### 2.7. Subcellular Localization and Protein–Protein Interaction Analysis

**Figure 8:**
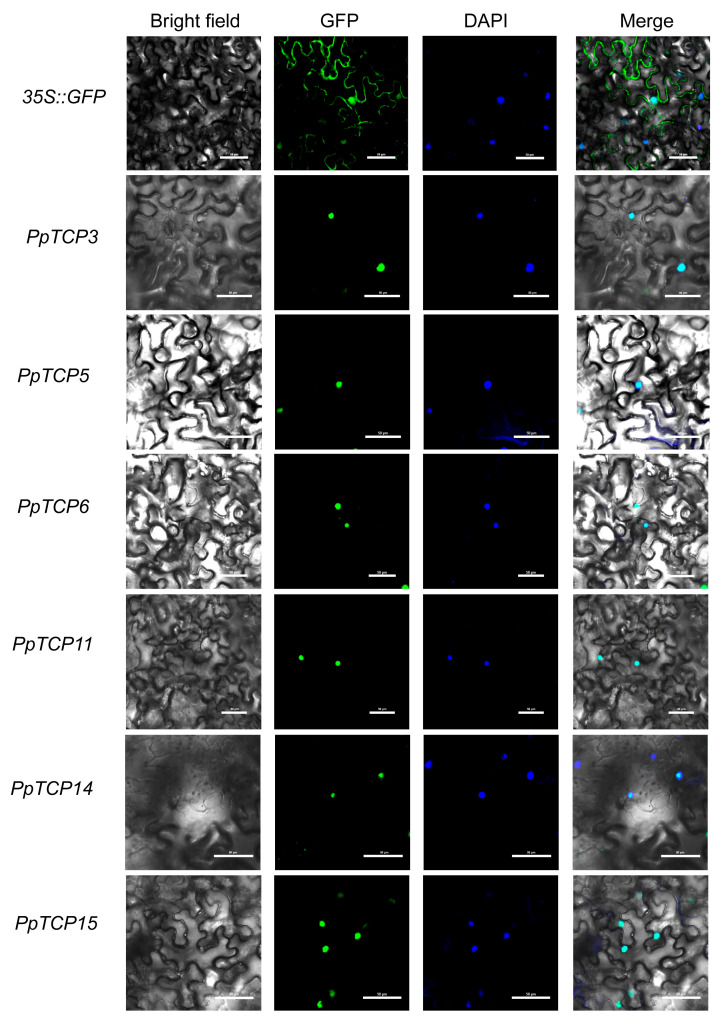
Subcellular localization of six PpTCP fusion proteins in *N. benthamiana* leaves. The GFP signal (green) represents the localization of the PpTCP-eGFP fusions, while DAPI (blue) marks the nuclei. Scale bars = 50 μm.

To determine the functional sites of the six putative candidate PpTCPs (*PpTCP3*, *5*, *6*, *11*, *14*, and *15*), we fused their coding sequences to the N-terminus of enhanced green fluorescent protein (eGFP) under the constitutive CaMV 35S promoter. These constructs were transiently expressed in *Nicotiana benthamiana* leaves via *Agrobacterium*-mediated transformation. This was followed by confocal microscopy, complemented by DAPI nuclear staining. As seen in Figure 8, *35S::GFP* was localized in the nucleus and cytoplasm. And the GFP signals emitted by the six PpTCP-eGFP fusion proteins were detected in the nucleus and overlapped with the DAPI staining. These results confirm that these transcription factors are localized to and function within the nuclear compartment.

**Figure 9:**
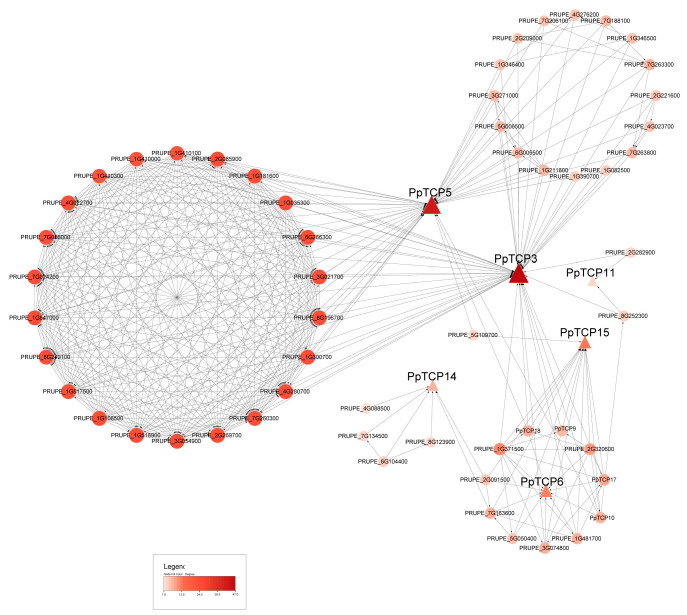
Protein–protein interaction (PPI) network for putative candidate PpTCPs. Interactions were retrieved from the STRING database (confidence score ≥ 0.500) and visualized in Cytoscape [62]. The six putative candidate PpTCPs are represented by triangular nodes. Node size and color intensity (from yellow to dark red) are proportional to the degree of connectivity, with larger, redder nodes indicating higher numbers of interacting partners.

To further explore the regulatory landscape of these putative candidates, we constructed a predicted protein–protein interaction (PPI) network using the STRING database [61] and visualized the results in Cytoscape 3.10.4. Analysis using the CytoHubba 0.1 degree algorithm revealed distinct topological roles for each protein (Figure 9; Table S3). Among the six putative candidates, PpTCP3 exhibited the highest connectivity with 47 predicted partners, and PpTCP5 was identified as another highly connected node. In contrast, PpTCP6, PpTCP14, and PpTCP15 were positioned within discrete, moderately connected modules, pointing toward potentially more specialized functions, whereas PpTCP11 showed the lowest connectivity with only two predicted partners. Table S4 presents a range of potential functional annotations for the predicted interacting proteins.

PpTCP3 emerged as the primary central hub with 47 predicted partners, suggesting it acts as a master regulator within a dense core signaling module. PpTCP5 was identified as a secondary prominent node, likely serving as a critical bridge that links the central core to peripheral subnetworks and facilitates crosstalk between different molecular complexes. In contrast, PpTCP6, 14, and 15 were situated within discrete, moderately connected modules, pointing toward more specialized, context-dependent functions. PpTCP11 exhibited the lowest connectivity with only two predicted partners. This tiered regulatory architecture provides a roadmap for future investigations into how TCP transcription factors coordinately govern peach development and environmental adaptation. Detailed functional annotations for the interacting proteins are provided in Table S4.

## 3. Discussion

While the TCP transcription factor family has been extensively characterized in diverse plant species, research in peach has largely been confined to its role in fruit ripening and bud endodormancy [44]. This leaves its broader involvement in vegetative development and stress adaptation largely unexplored. In this study, we leveraged the high-quality “RYP1” reference genome [4] to perform a comprehensive re-annotation and systematic characterization of the peach TCP family, specifically evaluating its transcriptional dynamics under drought stress. We identified 20 *PpTCP* family members, a count that aligns with previous reports [44] and confirms the stability of the TCP gene set across different peach genomic assemblies.

Phylogenetic reconstruction categorized the 20 PpTCP proteins into the two canonical plant classes: Class I (PCF clade) and Class II, with the latter further divided into CYC/TB1 and CIN sub-clades (Figure 2). This topology mirrors the conserved evolutionary structure observed in maize [63], suggesting that the regulatory logic of TCP genes has been strictly maintained across divergent plant lineages. Our analysis showed that *PpTCP* genes share the highest homology with those in apple and pear, reinforcing the close evolutionary proximity of these Rosaceae genera and their recent divergence from a common ancestor [64]. Furthermore, genome-wide collinearity analysis revealed higher synteny between peach and other *Rosaceae* species than with *Brassicaceae* or *Poaceae* (Figure 6).

Comparative genomic synteny analysis between peach and seven other species (*A. thaliana*, *S. lycopersicum, M. domestica*, *P. bretschneideri*, *P. armeniaca*, *O. sativa*, and *Z. mays*) further elucidated the evolutionary history of the *PpTCP* family (Figure 6). The higher frequency of *PpTCP* homologs within Rosaceae compared to Brassicaceae and Poaceae underscores the close phylogenetic proximity of these species. This functional conservation throughout evolution likely mirrors speciation patterns, suggesting that cross-species TCP research is vital for decoding plant evolutionary trajectories. Furthermore, physicochemical profiling indicated that all PpTCP proteins are hydrophilic (Table 1), possessing high hydration capacities [45,47]. This trait aligns with their demonstrated nuclear localization (Figure 8), facilitating their essential roles in transcriptional regulation. Similar nuclear-targeting patterns have been documented in *Gossypium hirsutum* [65], *B. platyphylla* [40], and sweet cherry [66].

Transcription factors (TFs) govern gene expression by binding to specific *cis*-acting elements [67], with the diversity of these sites dictating their regulatory scope [68]. Our analysis identified a broad spectrum of *cis*-regulatory motifs within the *PpTCP* promoters, including essential transcriptional elements (TATA and CAAT boxes) and various light-responsive motifs such as Sp1, Gap-Box, ACE, and GA-motifs (Figure 5A,B; Table S2). Additionally, the abundance of hormone-responsive elements across all 20 *PpTCP* genes (Figure 5A,B; Table S2) mirrors findings in legumes [69] and *Setaria italica* and *Setaria viridis* [70]. These observations raise the possibility that the PpTCP family may act as a potential hub integrating photosynthesis, hormonal signaling, and developmental adaptations to environmental stress. However, it should be emphasized that these inferences are derived from in silico promoter analysis and do not constitute direct evidence of transcriptional regulation by the corresponding hormones. Experimental validation is required to determine whether these cis-elements are functionally relevant in the context of stress responses. Notably, the promoters of PpTCP14—a rapidly drought-responsive candidate gene—lack ABREs, suggesting that its drought-induced expression may be mediated through ABA-independent pathways or via alternative regulatory elements not captured by current prediction algorithms.

Building on this structural foundation, we evaluated the transcriptional dynamics of the *PpTCP* family under simulated drought. Six members (*PpTCP3*, *5*, *6*, *11*, *14*, and *15*) exhibited significant differential expression, identifying them as primary drought-responsive putative candidates (Figure 7). To explore their underlying molecular mechanisms, we used the STRING database to construct a protein–protein interaction (PPI) network (Figure 9). The high connectivity of PpTCP3 and PpTCP5 within this network highlights their potential as master regulators. Functional annotation of these interacting partners (Table S4) revealed associations with key hormone pathways and stress-related TFs, including auxin signaling (*PRUPE_1G481700*, *3G074800*, *8G252300*) [71] and strigolactone signaling (*PRUPE_1G410000*) [72]. Notably, one interactor (*PRUPE_2G091500*) is an ortholog of the *Arabidopsis* stress-responsive NAC factor *ANAC078* [73]. These data also suggest that different TCP members may physically interact to coordinately regulate specific biological pathways.

In summary, the present findings provide a comprehensive characterization of the peach PpTCP gene family and offer preliminary insights into its potential involvement in drought-associated transcriptional responses. *PpTCP3* and *PpTCP5* may participate in the regulatory networks underlying stress adaptation. However, it should be emphasized that these inferences are derived from transcriptomic and in silico analyses, and further experimental validation is required to confirm their biological functions and regulatory mechanisms. Subsequent research endeavors will concentrate on elucidating the specific roles of these putative candidate genes in stress responses and their potential influence on fruit quality. These findings may serve as a foundation for identifying molecular targets that could contribute to the breeding of improved peach varieties.

## 4. Materials and Methods

### 4.1. Plant Materials and Drought Treatment

The peach cultivar ‘Rui Youpan 1’ (‘RYP1’), cultivated in Pinggu District, Beijing, China, served as the experimental material. Peach tree cuttings were collected during the first rapid growth stage. Cuttings bearing fruits of similar size were placed in Hoagland medium (Coolaber, Beijing, China) for overnight preculture. Subsequently, the precultured peach cuttings were transferred to Hogland medium containing 300 mM mannitol to establish osmotic stress conditions. Fruits were harvested from the branches at 0 h, 0.5 h, 1 h, 2 h, 4 h, 8 h, 12 h, 24 h, and 48 h after the 300 mM mannitol treatment and immediately stored at −80 °C until used for RT-qPCR analysis [61]. In the experiment simulating osmotic stress on fruit-bearing branches, three fruits were collected at each time point as biological replicates.

### 4.2. Identification and Physicochemical Characterization of PpTCP Genes

Candidate *TCP* genes in the ‘RYP1’ genome were identified using the Simple HMM Search tool in TBtools II [60], employing the TCP domain profile (PF03634) retrieved from the InterPro database. Predicted protein sequences were cross-validated using SMART v10 and NCBI-CDD v3.21; sequences lacking the characteristic TCP domain or identified as redundant were excluded. Ultimately, 20 *PpTCP* genes were confirmed. Physicochemical properties, including molecular weight and isoelectric points, were calculated using the Protein Pairwise Similarity Matrix tool in TBtools. Chromosomal coordinates for all *PpTCP* members were mapped based on ‘RYP1’ genomic annotation data.

### 4.3. Phylogenetic, Structural, and Conserved Domain Analysis

TCP protein sequences from *A. thaliana*, *O. sativa*, *S. lycopersicum*, *M. domestic* and *P. bretschneideri* were obtained from the Plant TFDB database. A Maximum Likelihood (ML) phylogenetic tree [50] was constructed using MEGA 7.0 [57], incorporating sequences from *A. thaliana* (AtTCPs), *O. sativa* (OsTCPs), *S. lycopersicum* (SlTCPs), *P. bretschneideri* (PbTCPs), *M. domestica* (MdTCPs), and *P. persica* (PpTCPs) with 1000 bootstrap replicates. The resulting phylogeny was visualized via iTOL 7.6 [74]. Gene architecture was characterized using the Visualize Gene Structure function in TBtools v2.486. Conserved domains were identified using NCBI Batch-CD-Search and visualized using the Domain Pattern tool in TBtools v2.486.

### 4.4. Multiple Sequence Alignment and Motif Discovery

Multiple sequence alignments of PpTCP proteins were performed in MEGA 7.0 and refined using GeneDoc 2.7 [75]. Conserved motifs were identified using the MEME suite, with the maximum number of motifs set to 10 and all other parameters at default settings [76]. Motif patterns were subsequently visualized using TBtools v2.486.

### 4.5. Promoter Cis-Acting Element Prediction and Collinearity Analysis

For each *PpTCP* gene, the 2000 bp genomic sequence upstream of the start codon (CDS) was extracted using the TBtools sequence toolkit. Regulatory *cis*-elements were predicted using PlantCare [77], followed by statistical filtering and visualization in TBtools. For collinearity analysis, genomic and annotation files for *A. thaliana* (TAIR), *O. sativa*, *Zea mays*, *S. lycopersicum*, *P. bretschneideri*, and *M. domestica* (NCBI) were utilized. Syntenic blocks and orthologous *TCP* pairs between these species and peach were identified using the One Step MCScanX-Super Fast algorithm and visualized via the Dual Synteny Plot function in TBtools.

### 4.6. Expression Profiling and RT-qPCR Validation

Transcriptomic data from peach fruits (0–48 h of drought time-course) were used to quantify *PpTCP* expression levels. Total RNA was extracted using the RNAprep Pure Plant Kit (TIANGEN, Beijing, China). RNA concentration, purity, and integrity were assessed using NanoDrop 2000 (Thermo Fisher Scientific, Waltham, MA, USA) and Agilent Bioanalyzer 2100 (Agilent Technologies, Santa Clara, CA, USA). For RNA-seq, 27 cDNA libraries (three biological replicates per nine time points) were constructed using the Hieff NGS Ultima Dual-mode mRNA Library Prep Kit for Illumina (Yeasen Biotechnology, Shanghai, China) and sequenced on the Illumina NovaSeq platform in PE150 mode. After quality filtering, 168.73 Gb of clean data was generated, with Q30 ≥ 93.35% for all samples. Clean reads were aligned to the peach reference genome (*Prunus persica* v2.0.a1.genome.fa) using HISAT2 2.2.1 tools soft [78], with mapping ratios of 93.12–94.89%. Gene expression was quantified as FPKM using StringTie [79]. Differential expression analysis was performed using DESeq2 [80] with |log_2_FC| ≥ 1 and FDR < 0.01 as the threshold. We performed sequencing using Biomarker Technologies Co., Ltd. (Beijing, China). RT-qPCR primers (Table S5) were designed via NCBI Primer-BLAST (https://blast.ncbi.nlm.nih.gov/Blast.cgi, accessed on 16 July 2026) and *PpActin* (*Prupe.6G163400*) was selected as the reference gene. Reactions (20 μL) contained 2 μL cDNA, 0.4 μL of each primer (10 μM), 10 μL Universal SYBR qPCR Master Mix (Vazyme), and 7.2 μL ddH_2_O. Three biological replicates and four technical replicates were performed per sample. Relative expression was calculated using the 2^−△△Ct^ method with statistical significance determined by one-way ANOVA multiple comparisons in GraphPad Prism 8.0.

### 4.7. Subcellular Localization and PPI Network Prediction

The CDS of each selected *PpTCP* (excluding the stop codon) was cloned into the pCAMBIA-super-1300-eGFP vector under the control of the CaMV 35S promoter. Constructs were verified using SnapGene V6.0.2 [81]. *A. tumefaciens* strain GV3101 carrying either the recombinant or empty vector was infiltrated into 5-week-old *N. benthamiana* leaves using an infiltration buffer (200 mM MES, pH 5.6; 1 M MgCl_2_; 100 μM acetosyringone) [82].

Plants were kept in darkness overnight, followed by 24 h of low-light cultivation. GFP signals and DAPI-stained nuclei were visualized using a Nikon A1 confocal laser scanning microscope (Nikon, Tokyo, Japan) and analyzed with NIS-Elements Viewer 5.21. Finally, protein–protein interaction (PPI) networks were predicted using STRING 12.0 and visualized in Cytoscape [62].

## Figures and Tables

**Table 1:** Molecular characteristics of *PpTCP* genes in peach.

Gene ID	Name	Number of Amino Acids	Molecular Weight (KD)	Instability Index	Aliphatic Index	Hydropathicity
RYP1_01G00661	PpTCP1	281	29.57	46.10	58.04	−0.68
RYP1_01G02200	PpTCP2	267	28.49	53.19	69.06	−0.51
RYP1_01G02701	PpTCP3	425	44.99	55.58	59.79	−0.62
RYP1_01G02997	PpTCP4	504	56.14	47.72	54.40	−0.93
RYP1_01G05584	PpTCP5	603	63.10	70.64	51.44	−0.76
RYP1_02G01559	PpTCP6	296	31.62	56.59	70.00	−0.39
RYP1_02G02812	PpTCP7	382	39.73	46.78	62.91	−0.43
RYP1_03G01090	PpTCP8	192	20.90	43.90	66.61	−0.69
RYP1_03G02271	PpTCP9	424	47.49	56.83	63.51	−0.76
RYP1_03G02400	PpTCP10	376	41.41	49.10	65.66	−0.78
RYP1_03G02931	PpTCP11	311	33.48	64.20	63.41	−0.76
RYP1_04G00552	PpTCP12	286	32.50	74.06	58.36	−1.02
RYP1_04G00796	PpTCP13	500	54.39	53.00	56.48	−0.91
RYP1_04G01613	PpTCP14	439	47.77	48.74	58.29	−0.70
RYP1_05G00875	PpTCP15	373	38.84	53.40	62.79	−0.47
RYP1_05G01851	PpTCP16	414	44.35	64.42	55.19	−0.73
RYP1_05G01987	PpTCP17	381	42.18	53.80	55.80	−0.89
RYP1_05G02096	PpTCP18	468	53.62	59.73	60.64	−0.98
RYP1_06G02286	PpTCP19	227	24.77	68.93	71.89	−0.61
RYP1_07G01014	PpTCP20	463	50.54	52.26	86.16	−0.20

## Data Availability

The raw data supporting the conclusions of this article will be made available by the authors on request.

## Supplementary Materials

The following supporting information can be downloaded at: https://www.mdpi.com/article/10.3390/ijms27156860/s1.

## Abbreviations

**Abbreviation**	**Full Name**
ABA	Abscisic acid
ABRE	ABA-responsive element
bHLH	Basic helix–loop–helix
CaMV	Cauliflower mosaic virus
CDS	Coding sequence
CIN	CINCINNATA (TCP class II subclade)
CYC/TB1	CYCLOIDEA/TEOSINTE BRANCHED 1 (TCP class II subclade)
eGFP	Enhanced green fluorescent protein
GRAVY	Grand average of hydropathicity
MeJA	Methyl jasmonate
MEME	Multiple EM for Motif Elicitation
ML	Maximum likelihood
PCF	PROLIFERATING CELL FACTOR (TCP class I)
PPI	Protein–protein interaction
RT-qPCR	Reverse transcription quantitative polymerase chain reaction
RNA-seq	RNA sequencing
STRING	Search Tool for the Retrieval of Interacting Genes/Proteins
TCP	Teosinte branched 1/Cycloidea/Proliferating cell nuclear antigen factor
